# The amino acid selected for generating mutant TbpB antigens defective in binding transferrin can compromise the *in vivo* protective capacity

**DOI:** 10.1038/s41598-018-25685-1

**Published:** 2018-05-09

**Authors:** João Antônio Guizzo, Somshukla Chaudhuri, Simone Ramos Prigol, Rong-hua Yu, Cláudia Cerutti Dazzi, Natalia Balbinott, Gabriela Paraboni Frandoloso, Luiz Carlos Kreutz, Rafael Frandoloso, Anthony Bernard Schryvers

**Affiliations:** 1 Laboratory of Microbiology and Advanced Immunology, Faculty of Agronomy and Veterinary Medicine, University of Passo Fundo, Passo Fundo, 99052-900 Brazil; 2 Department of Microbiology & Infectious Diseases, Faculty of Medicine, University of Calgary, Calgary, Alberta T2N 4N1 Canada

## Abstract

*Haemophilus parasuis* is the causative agent of the Glässer’s disease (GD), one of the most important bacterial diseases that affect young pigs worldwide. GD prevention based on vaccination is a major concern due to the limited cross-protection conferred by the inactivated whole cell vaccines used currently. In this study, vaccines based on two mutant recombinant proteins derived from transferrin binding protein B of *H. parasuis* (Y167A-TbpB and W176A-TbpB) were formulated and evaluated in terms of protection against lethal challenge using a serovar 7 (SV7) *H. parasuis* in a high susceptibility pig model. Our results showed that *H. parasuis* strain 174 (SV7) is highly virulent in conventional and colostrum-deprived pigs. The Y167A-TbpB and W176A-TbpB antigens were immunogenic in pigs, however, differences in terms of antigenicity and functional immune response were observed. In regard to protection, animals immunized with Y167A-TbpB antigen displayed 80% survival whereas the W176A-TbpB protein was not protective. In conjunction with previous studies, our results demonstrate, (a) the importance of testing engineered antigens in an *in vivo* pig challenge model, and, (b) that the Y167A-TbpB antigen is a promising antigen for developing a broad-spectrum vaccine against *H. parasuis* infection.

## Introduction

*Haemophilus parasuis* (Hps) is the causative agent of the Glässer’s disease, one of the most globally important bacterial diseases of swine, which affects piglets mainly in the nursery phase of production. Virulent strains of *H. parasuis* causes a systemic infection characterized by polyarthritis, polyserositis and meningitis^1^, leading to important economic losses for the pig industry due to mortality and the expensive treatment of sick animals.

Isolates of *H. parasuis* from invasive infection express an extracellular polysaccharide capsule, which forms the basis for a serotyping (serovar) classification scheme in which sera only reacts with a specific capsular type. Recently the capsular loci from the 15 recognized serovars were sequenced^2^ and used to develop a multiplex PCR (mPCR) method for rapid molecular typing of *H. parasuis*^3^. Although some reports showed that all clinical isolates could be serotyped by mPCR^3,4^, others have highlighted the existence of a number of nontypeable clinical isolates from GD outbreaks^5,6^, suggesting that there may be new capsular types not yet characterized and could indicate that there may be a repertoire of capsular types available to *H. parasuis*.

Inactivated whole cell vaccines (bacterins) have been the predominant approach for prevention of *H. parasuis* infection. Since the preparation of bacterins closely resembles the preparations used for generating sera for serotyping, they logically will primarily induce antibodies that target the polysaccharide capsule. As experience with conjugate capsular vaccines has clearly demonstrated, the protection against capsular polysaccharide is limited to strains expressing that specific capsular polysaccharide. Thus, these bacterins are expected to provide protection against strains expressing the same type of polysaccharide capsule but may be limited in their ability to induce cross-protective antibodies. The efficient horizontal exchange provided by natural transformation can result in transfer of virulence determinants^7^ or capsule switching^8,9^, altering the prevalence of capsular types in disease isolates over time^10^. Thus, it may be necessary to monitor the prevalence of *H. parasuis* capsular types in different geographical regions and adjust the composition of bacterins in order to maintain vaccine efficacy.

The potential limitation of inactivated whole cell vaccines has led to efforts at developing protein-based vaccines that include Omp16^11^, SOD^12^ and HxuC^13^ in which the recombinant antigens have been shown to protect mice against lethal challenge with *H. parasuis*. Recombinant antigens that include GAPDH-OapA-HPS-0675^14^, VtaA^15^ and OppA^16^ have been tested in a pig infection model with variable protection against homologous challenge. Unfortunately, there is little information on the cross-reactive and cross-protective properties of the immune response induced by commercial or experimental bacterins or the protein subunit vaccines^17^.

To overcome the limited protection conferred by current vaccines, we explored the potential of transferrin binding protein B (TbpB), a surface glycoprotein involved in acquiring iron from host transferrin (Tf), as a potential vaccine candidate. The recognition that the native TbpB protein might be complexed with host Tf during systemic administration, potentially blocking important epitopes required for protection, prompted us to implement a structure-based design approach to generate TbpBs defective in binding porcine Tf. One of the engineered mutant TbpBs, Y167A, had a 300-fold reduction in binding affinity relative to native TbpB, and induced a more protective immune response than native TbpB against infection by *H. parasuis* in colostrum-deprived pigs^18^. This vaccine provided complete protection against infection and was shown to induce a strong T helper 2 response^19^, a high percentage of B cells in the peripheral blood after immunization^18^ and antibody with wide cross-reactivity against different virulent strains of *H. parasuis*^20^.

The TbpB proteins are also present in two other important porcine pathogens, *Actinobacillus pleuropneumoniae* and *A. suis*. Analysis of the sequence and structural diversity of the TbpB proteins in the three species (*H. parasuis, A. pleuropneumoniae* and *A. suis*) demonstrated that the sequences cluster independently from species, geographical region or time of isolation, indicating that there has been extensive genetic exchange between these species and suggesting that most variants have not arisen recently^21^. Since ready exchange of variant genes may be the primary mode of immune evasion, it would be logical for a TbpB-based vaccine to target all three species. Furthermore, analysis of the antibody response against a wild-type TbpB from *A. pleuropneumoniae* demonstrated high levels of cross-reactivity against a TbpB from the same phylogenetic cluster, suggesting that a vaccine comprised of a limited number of TbpB antigens would likely be broadly cross-protective^21^.

To further explore the potential for developing a broadly cross-protective vaccine targeting TbpB it was important to determine whether generation of mutants that substantially reduce binding of Tf is sufficient to predict a protective immune response capable of preventing infection. Thus, in this study, we compare the ability of two mutant TbpBs defective in binding transferrin to protect against infection in the *H. parasuis* pig infection model. Various immunological parameters were measured in an attempt to evaluate their contribution to protection from infection.

## Results

### Sequence Analysis and Selection of TbpB Variants

It is important to recognize that due to efficient exchange of variant genes through natural transformation inherent in the targeted porcine pathogens^22^, the TbpB variant is unlikely to be tightly linked to capsular type or perhaps even to a dominant virulent strain or lineage. In addition, it is certainly possible that a virulent serovar or strain could alter its TbpB variant or acquire a different variant during a disease outbreak in a geographical region or possibly even when a strain is passaged through pigs to increase its virulence. We observed that the sequences of two TbpBs from the genomic sequences of Nagasaki strains in the NCBI database differed, confirming that assumptions cannot be made regarding TbpB variant in a particular lineage.

**Figure 1:**
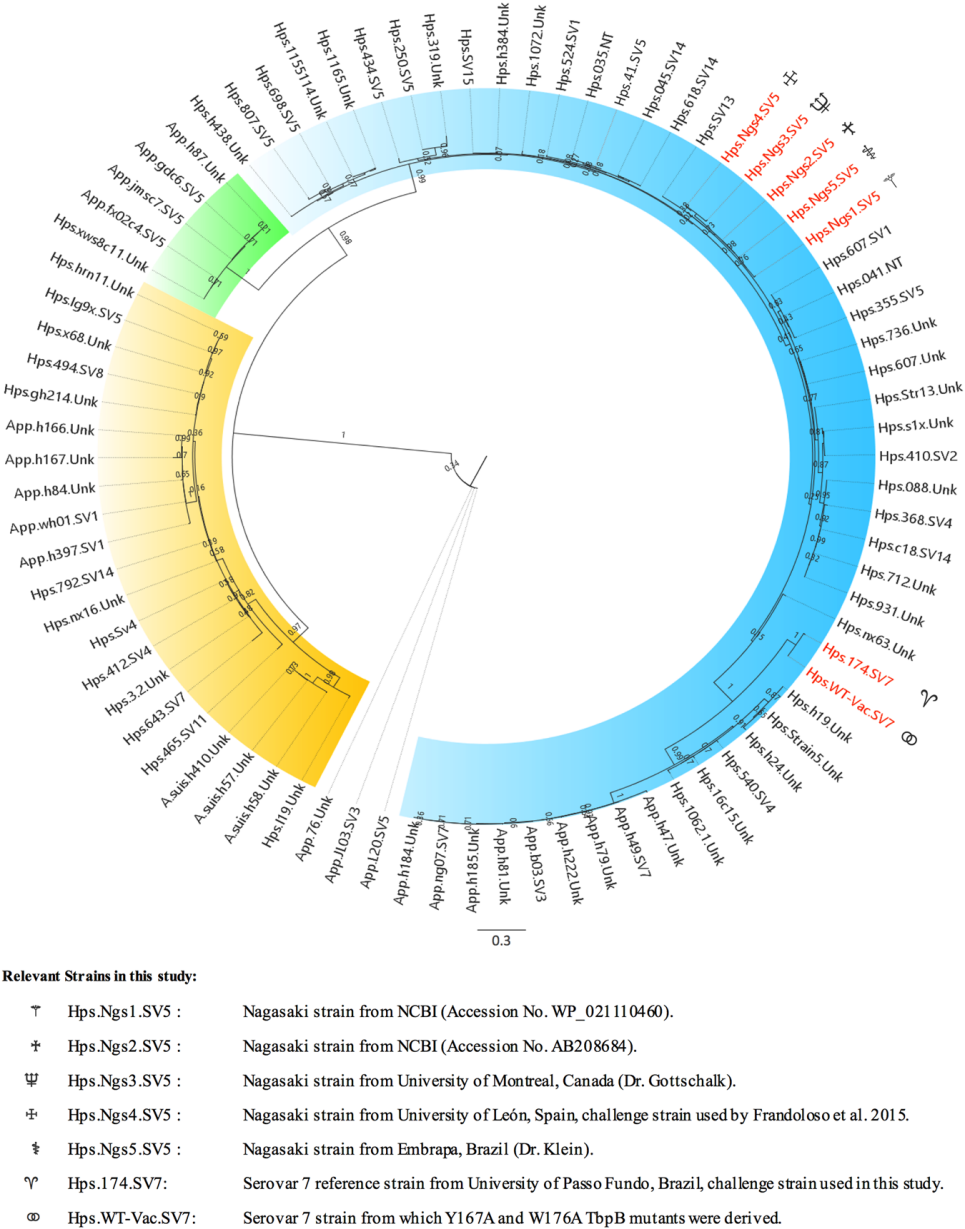
Sequence diversity of TbpBs from porcine pathogens. Maximum likelihood tree demonstrating the overall diversity of TbpBs from *H. parasuis, A. pleuropneumoniae* and *A. suis*. Leaf labels identify the strains from which TbpB sequences were obtained and indicate their species and serovar, if known (NT = Nontypeable, Unk = Unknown). The sequences are rooted by the three sequences with a white background, which are the secondary TbpB-like genes. Sequences of interest to this study are labelled in red. The sequences clustered into three main groups (Group 1 = yellow background, Group 2 = green background, Group 3 = blue background) with high confidence. The branch support values are displayed.

This issue prompted us to continue to sequence the genes from our expanding collection of strains, and confirm the sequence of TbpBs in the strains used in our challenge experiments. As illustrated in Fig. 1, the sequences of our expanded collection of isolates still clustered into three main groups that were largely independent of species and serovar. Notably, the TbpBs from the genomic sequences of five Nagasaki strains (two from the NCBI database and three from different laboratory collections) had minor variations in sequence. Similarly, the TbpB variant from which the Y167A and W176A were derived is nearly identical to the TbpB present in *H. parasuis* strain 174, the challenge strain used in this study. However, it is from a different sub-cluster in Group 3 than the TbpB present in the Nagasaki challenge strain used in our previous study^18^. The observation that this antigen was able to provide complete protection from infection is even more impressive since it was a heterologous challenge experiment. The two TbpB variants belong to different clusters within the same group, indicating that this antigen may be able to provide cross-protection against any strain expressing a TbpB variant in Group 3. We anticipate that mutant TbpBs from Group 1 and Group 2 would also be able to provide cross-protection within their group, which provides support for the original proposal that the observed cross-reactivity of the antibody response suggested that a limited number of representative antigens could induce a cross-protective response against all variants^21^.

### Preparation of Vaccine Formulations

A primary goal of this study was to evaluate two different versions of an engineered mutant TbpB for their ability to induce a protective immune response against experimental infection by *H. parasuis*. Since we had previously shown that a non-binding mutant of TbpB provided superior protection against infection compared to wildtype TbpB^18^, we felt that it was important to determine whether the lack of binding was the primary or sole characteristic responsible for the dramatically enhanced protection. Thus, we decided to compare the Y167A mutant that was previously shown to induce an enhanced protective immune response to a similar conservative mutant from the same TbpB, W176A, that had a similar loss in binding (from K_d_ of 13 nM to K_d_ of 0.94 µM vs 3.64 µM, respectively). However, in contrast to the previous study, the recombinant proteins were not initially produced as fusions with an N-terminal tagged maltose-binding protein. Thus, the purified mutant TbpBs contain an N-terminal polyhistidine region that was used for purification with the Ni-NTA column in contrast to the TbpBs in the previous study that only contained a N-terminal glycine and serine that remained after removal of the N-terminal polyhistidine and maltose binding protein by cleavage with TEV protease.

Due to our recent analysis of the impact of adjuvant on the immune response in mice^20^ we opted to use Montanide Gel 01 PR instead of the Montanide IMS 2215 VR PR adjuvant used in the previous immunization and challenge experiment^18^ for preparation of the vaccine formulations. Prior to the challenge experiment the vaccine formulations were tested for sterility and stability *in vitro* and safety *in vivo*. No antigen precipitation, aggregation or color changes were observed in the formulated vaccines after storage for 30 days at 4 °C. Mice were immunized with the vaccines listed in Table 1 and clinically evaluated. All vaccines were shown to be completely safe; no mice died during the experiment, and no clinical abnormalities in mental state, behavior, body temperature, respiration, feces, skin, eyes and ear were observed in the treated mice.

**Table 1:** Experimental design of animal immunization.

Antigen	Pigs (*n*)	Dose	Adjuvant	Route	Immunization days	Serum collection days	PBMC collection days
Y167A TbpB	5	200 μg	Gel 01^b^	IM^a^	0, 21	0, 14, 21, 28, 35	0, 21, 24, 28, 35
W176A TbpB	5	200 μg	Gel 01	IM	0, 21	0, 21, 24, 28, 35	0, 21, 24, 28, 35
PBS-Gel 01	5	—	Gel 01	IM	0, 21	0, 21, 24, 28, 35	0, 21, 24, 28, 35

^a^Intramuscular;

^b^Montanide Gel 01 (final concentration of 20%).

### Analysis of the immune response to the formulated vaccines

**Figure 2:**
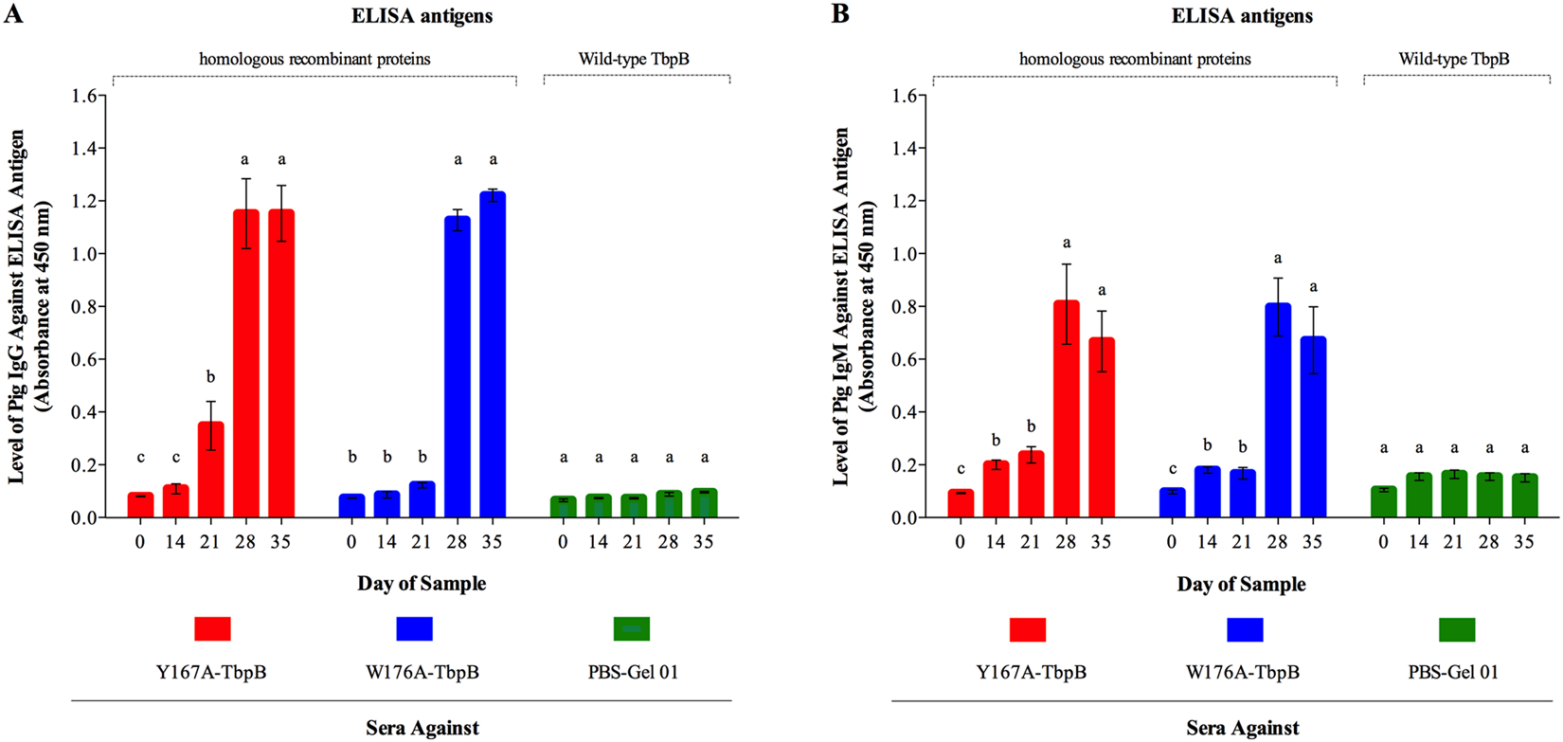
Kinetics of the specific antibody response in pigs. Sera collected from different time periods during the immunization schedule were diluted 1:100 and assessed by qualitative indirect ELISA. IgG responses are illustrated in the panel (A) and the IgM responses in the panel (B). Different letters indicate significant differences (p < 0.05) between the different time periods in a given group.

**Figure 3:**
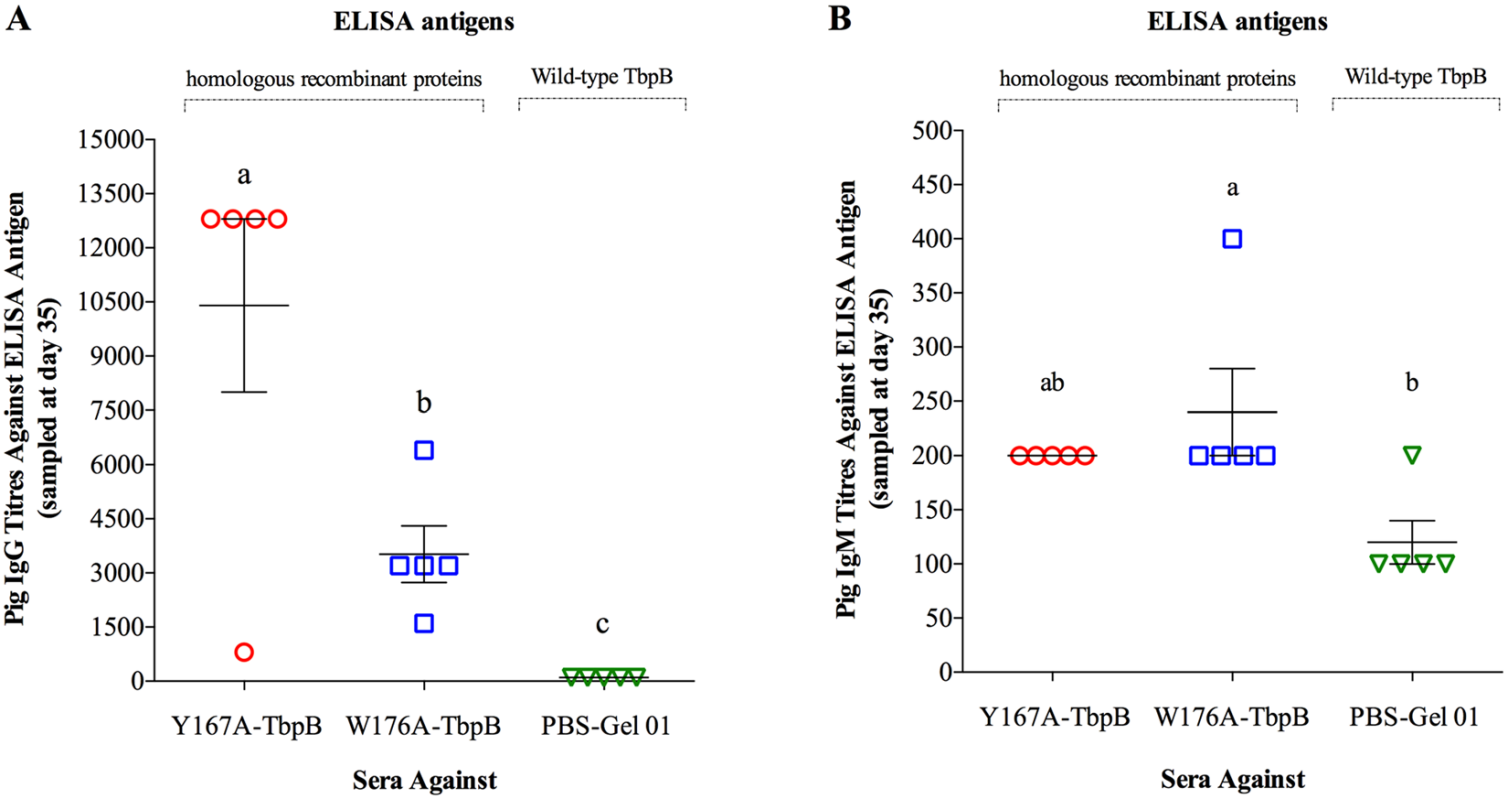
Antibody titres just prior to challenge (day 35). Sera collected at day 35 were titrated by two-fold serial dilution and assessed by indirect ELISA. (**A**) Titres of total sera IgG and (**B**) titres of total sera IgM. Different letters indicate significant differences (p < 0.05) between the different groups.

Prior to challenge the pigs were immunized with the different vaccine formulations at day 0 and day 21 by intramuscular injection and blood samples were taken on days 0, 14, 21, 28 and 35 for evaluation of the immune response (Fig. 2). Although not a quantitative measure of the magnitude of the antibody (IgG and IgM) response, the A_450_ attained with a 1:100 dilution of sera provided a comparison of the kinetics of the IgG and IgM responses against the two different antigens. Notably, there was a relatively low IgG response observed with the W176A-TbpB at day 21, prior to the second immunization. However, the 1:100 dilution of sera from pigs immunized with the two mutant TbpBs reached the maximal absorbance with the reagent for IgG by days 28 and 35, indicating that significant titres were achieved after the second immunization (Fig. 2A). In regards to the IgM response, both antigens were capable of inducing a significant absorbance increase after the first immunization (day 14), however, the maximum response was achieved at day 28, after the second immunization (Fig. 2B). Titres of sera for the individual groups at day 35 is illustrated in Fig. 3, in which the IgG titre induced by the vaccine formulated with W176A-TbpB was significantly less than that induced by the Y167A-TbpB (Fig. 3A). The titre of IgM at this time was low and similar in both immunized groups (Fig. 3B).

**Figure 4:**
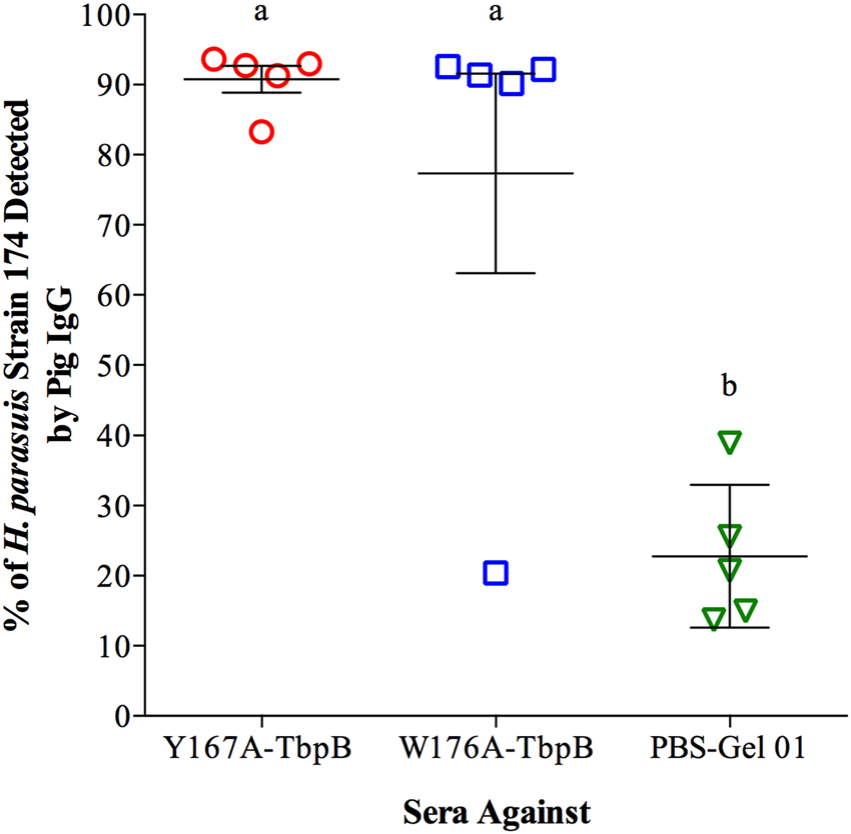
Ability of induced antibody to recognize intact *H. parasuis* cells. Heat treated sera collected at day 35 were incubated with live *H. parasuis* strain 174 (SV7) and analysed by flow cytometry. Different letters indicate significant differences (p < 0.05) between the different groups.

The analysis in Figs 2 and 3 measure the antibody response to the immunizing antigens, but does not evaluate the degree to which they can react with native epitopes present at the surface of the *H. parasuis* cells. Since bacteria are subjected to an iron limited environment in the host, *H. parasuis* strain 174 (SV7) was grown in the presence of the iron chelator desferoxamine to enhance the ability to recognize antigens expressed under *in vivo* conditions. FACS analysis was performed to measure reactivity with surface epitopes. As illustrated in Fig. 4, IgGs directed against the recombinant TbpB antigens (Y167A and W176A) were capable of recognizing virtually all of the *H. parasuis* cells, with the exception of one animal immunized with W176A-TbpB (Fig. 4).

**Figure 5:**
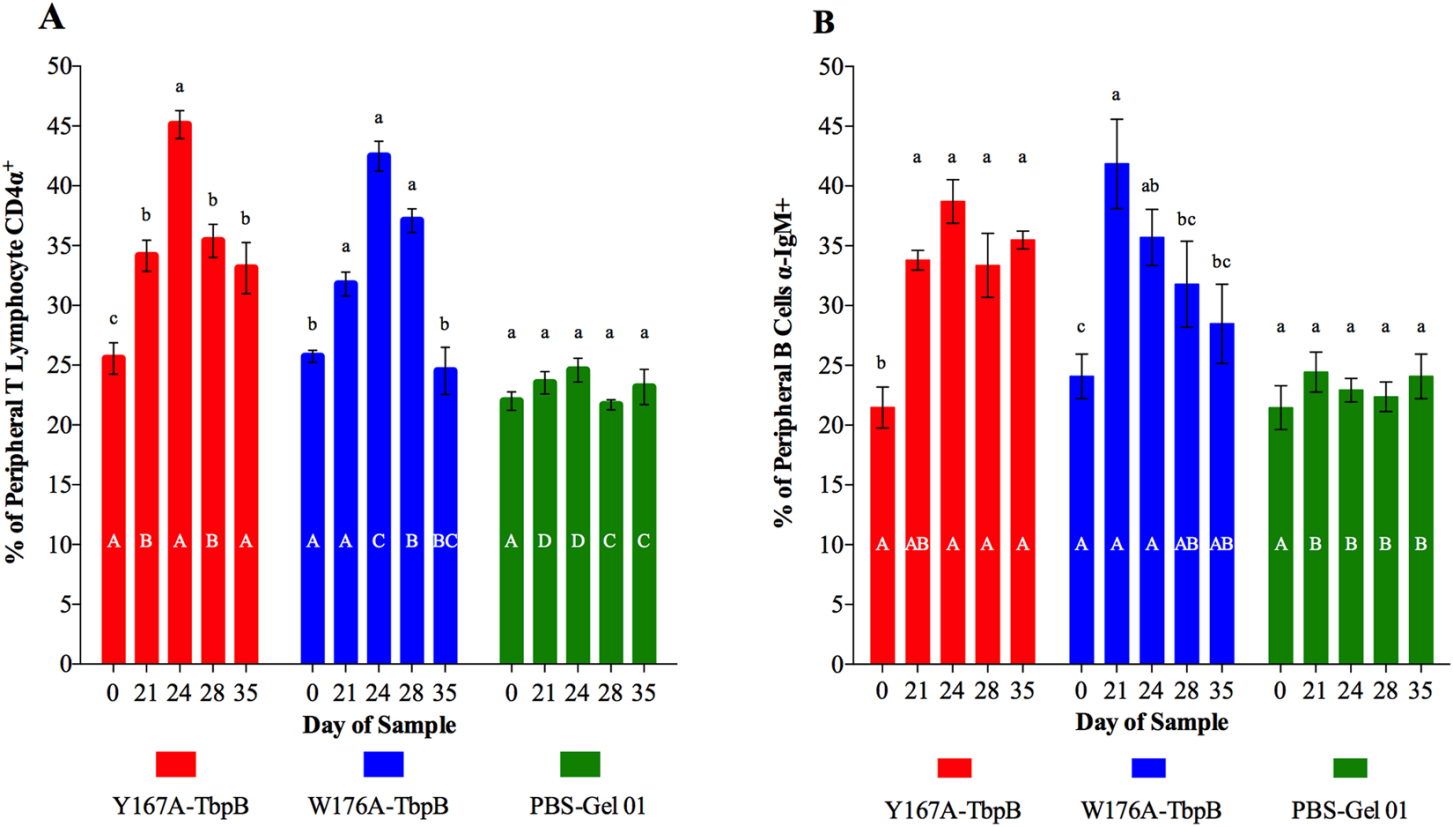
Cellular immune response during the vaccination protocol. Peripheral T lymphocytes CD4α+ (**A**) and B cells α-IgM+ (**B**) were analysed by flow cytometry before and after each immunization. Results are expressed as cell percentage relative to the total cells gated into the lymphocytes region (FSC vs SSC). Lower case letters indicate significant differences (p < 0.05) between the different days within the group. Capital letters indicate differences between the same days for different groups.

In parallel with evaluating the humoral (IgG) response in sera collected at the different time points (days 0, 14, 21, 28, 35), we measured the peripheral T-cell and B-cell responses from samples taken at days 21, 24, 28 and 35 (Fig. 5). Our results showed that all animals that received the recombinant antigens along with the Montanide Gel 01 adjuvant had significantly increased the percentage of CD4^+^ T cells after the first immunization (Fig. 5A), which indicates that all antigens promote the differentiation and maturation of T cells. After the second immunization (day 21), a fast-proliferative response (day 24) was observed in the peripheral blood of the animals immunized with Y167A-TbpB and W176A-TbpB (Fig. 5A).

All the pigs immunized with the mutant TbpBs had an increased B-cell response after the first immunization (day 21, Fig. 5B). To evaluate if the vaccine booster (day 21) could stimulate the B memory cells and increase its percentage relative to the day 21, we conducted a FACS analysis at three subsequent time points. A slight increase (~5%) on day 24 was only observed in those animals that received Y167A-TbpB and after that the percentage remained high (~38%) until day 35. Interestingly, animals immunized with W176A-TbpB displayed progressive reduction of the cellular response after the second immunization, reaching on day 35 a lower percentage (~28%) in comparison with the Y167A-TbpB vaccinated group (Fig. 5B).

**Figure 6:**
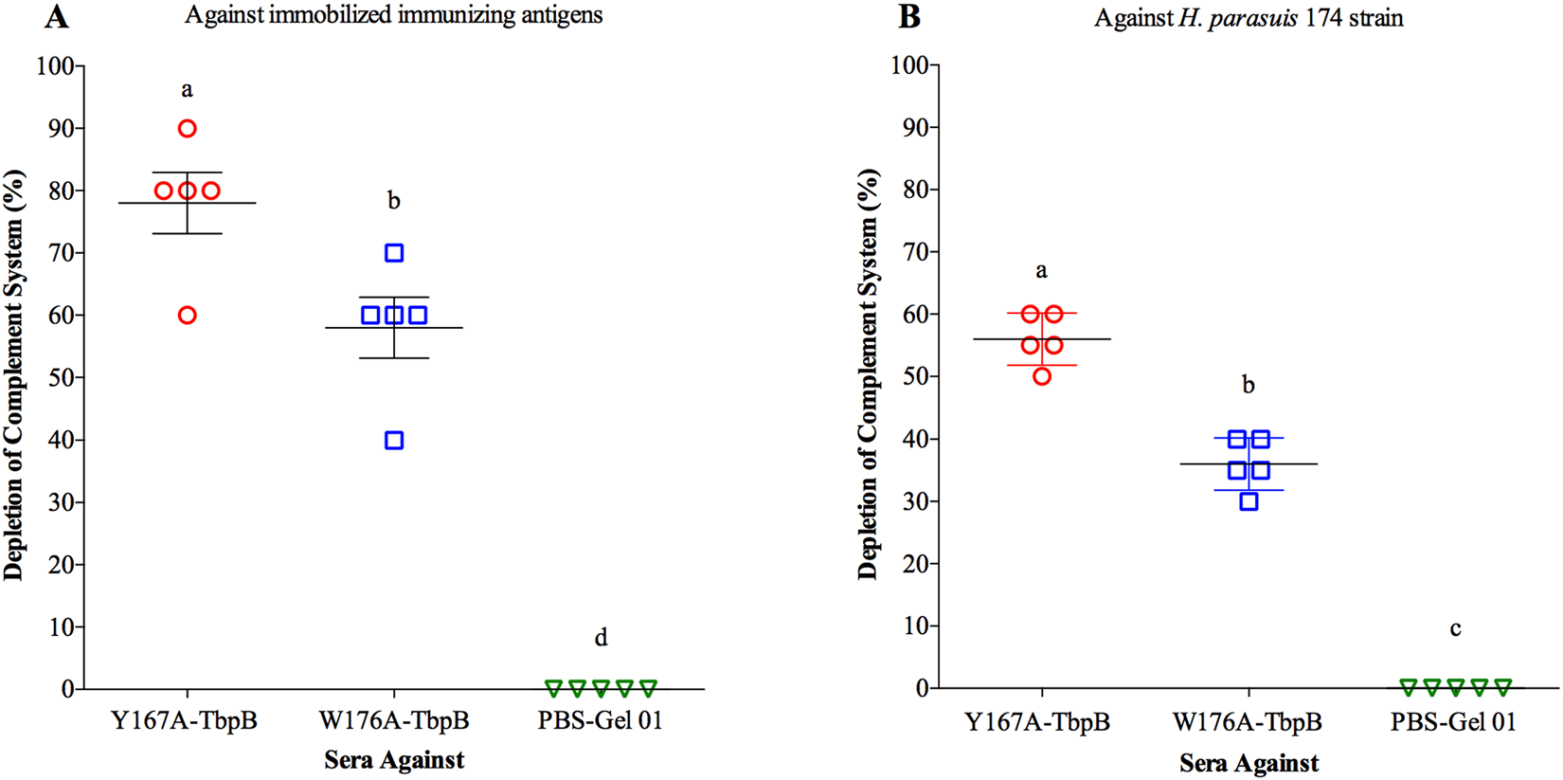
Activation of the classical pathway of the complement system. Heat inactivated sera from pigs immunized with 6 different vaccines were mixed with immobilized immunizing antigens (**A**) or live *H. parasuis* 174 strain (SV7) (**B**) to deplete complement, and the residual complement measured by erythrocyte lysis. The capacity of these induced antibodies to activate the complement system (deplete complement) is represented as percentage of activation. Different letters indicate significant differences (p < 0.05) between the different groups.

In an attempt to evaluate the functional capacity of the antibodies induced by the different vaccine formulations, the ability of inactivated sera to activate the classical complement system was assessed as previously described by our group^20^. With the exception of animals immunized with PBS-Gel 01, all other immunized pigs developed antibodies capable of activating the complement system as illustrated in Fig. 6. The highest activation level against the immunizing antigens was observed in the group that received the Y167A-TbpB version (Fig. 6A). A similar trend was observed against the *H. parasuis* 174 strain (Fig. 6B). These results reflect the difference in titres of IgG in the sera against the two mutant TbpBs (Fig. 3).

**Figure 7:**
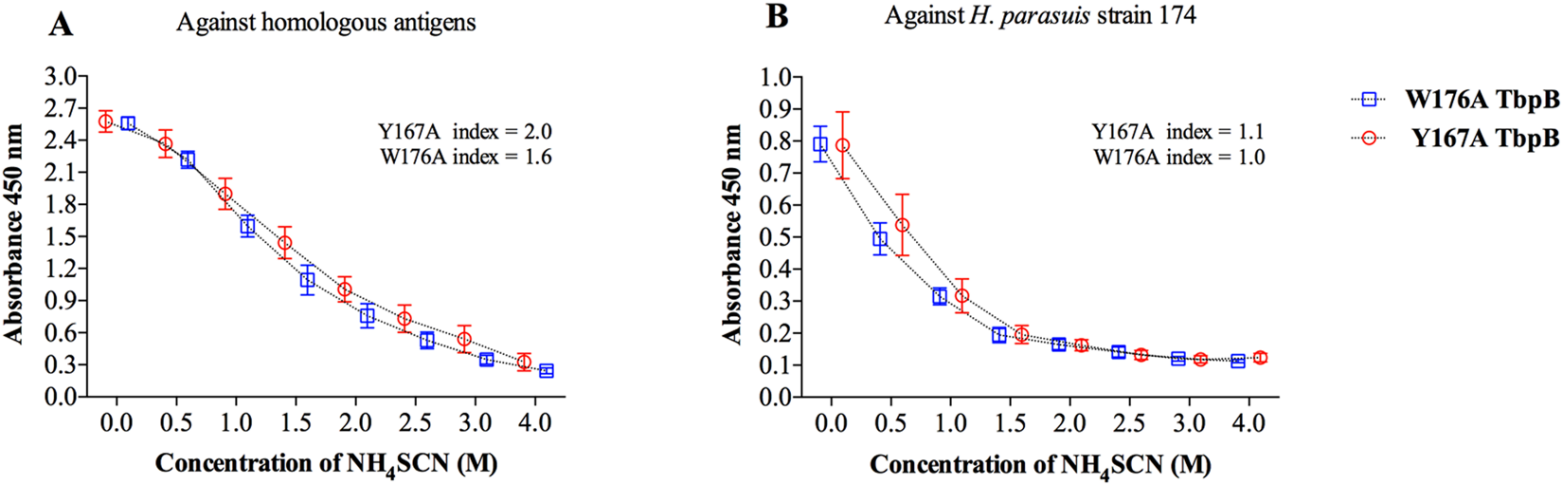
Avidity curves of polyclonal pig antibodies. The antibody binding force was measured by a thiocyanate elution assay. The avidity index is equal to the concentration of NH_4_SCN that gave 50% reduction in absorbance.

Because the avidity is known to influence the antibody effector functions, we measured the functional affinity of the pig polyclonal antibodies induced by the two-different versions of the mutant TbpB. When the antibody interaction was assessed against the homologous antigens, the IgGs induced by the Y167A-TbpB antigen showed a slightly higher avidity index than those generated by W176A-TbpB (2 and 1.6 M of NH_4_SCN, respectively) (Fig. 7A). A similar avidity trend was observed against the challenged strain (Fig. 7B).

### Immunization and challenge experiment with vaccine formulations

**Figure 8:**
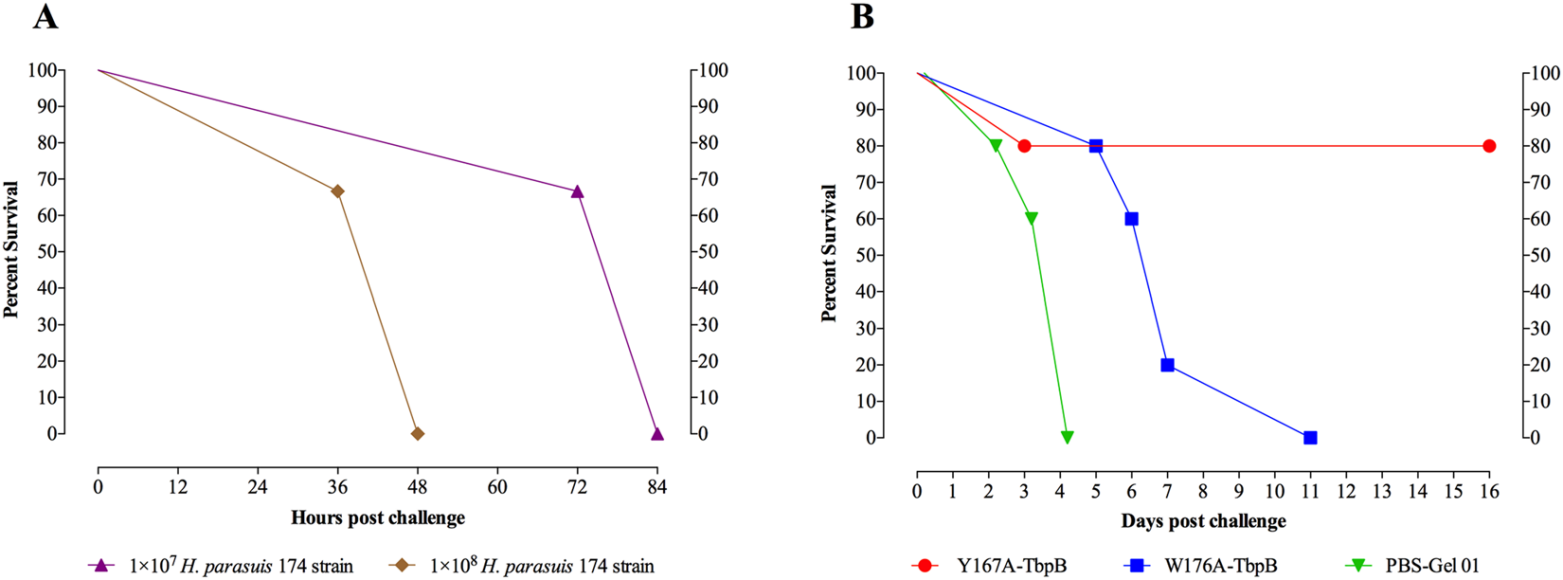
Survival rates of pigs challenged with *H. parasuis* 174 strain. (**A**) Evaluation of two dose of *H. parasuis* 174 strain [1 × 10^7^ (*n* = 3) or 1 × 10^8^ (*n* = 3)] in conventional pigs. (**B**) Comparative capacity of 2 experimental vaccines to induce a protective immune response against *H. parasuis* infection. Groups of pigs were immunized with PBS-Gel 01 (control), Y167A-TbpB and W176A-TbpB. The pigs were challenged by intratracheal inoculation with 1 × 10^7^ *H. parasuis* 174 strain and were monitored for clinical signs and symptoms throughout the duration of the experiment, which was 16 days.

Since there have been recent Glässer’s disease (GD) outbreaks by serovar 7 *H. parasuis* in Brazil (field strains isolated by Laudo Laboratório Avícola Uberlândia Ltda, Minas Gerais – Brazil, and typed by our laboratory) and in China^6^, we decided to consider performing the immunization and challenge experiment with a serovar 7 strain. Although it had been reported that the reference serovar 7 *H. parasuis* strain (174) was not virulent, we have observed that passage through pigs can generate a more virulent form of a strain suitable for challenge experiments. Thus, pigs were challenged with 1 × 10^7^ or 1 × 10^8^ bacteria by intratracheal injection with the intent of increasing the dose if disease was not observed. However, all animals died or were euthanatized between 36 and 84 hours post challenge (Fig. 8A). The disease evolution (fever, apathy, dyspnea, swollen joints and lameness) was faster in the animals challenged with the high dose than those that received the lower dose. During the necropsy, classical gross lesions characterized by severe fibrinous polyserositis in the pericardial, pleural, and peritoneal cavities were observed and *H. parasuis* was recovered from lungs, pleural, pericardial and peritoneal cavities, hock joints and brain, with exception of the animals that died at 36 hours that showed no bacteria in the brain. The bacteria recovered from the swab collected from the brain surface was grown on chocolate agar and then small aliquots of the culture were prepared and kept at −80 °C for the use in the immunization and challenge experiment.

In order to evaluate if the two experimental vaccines were capable of inducing a protective immune response against a lethal dose of *H. parasuis* 174 strain, colostrum-deprived pigs were challenged with 1 × 10^7^ *H. parasuis* by intratracheal injection (Fig. 8B). Four of the 5 pigs immunized with Y167A-TbpB survived for 16 days post challenge (experimental endpoint), achieving 80% protection, while all the pigs immunized with W176A-TbpB died or were euthanized by day 11. All the pigs belonging to the control group (PBS-Gel 01) died or were euthanized by day 4 after challenge (Fig. 8B). The rectal temperatures and the clinical symptom records are described in the Supplementary Fig. S1 and Table S1.

## Discussion

Currently the mainstay for prevention of Glässer’s disease is commercial vaccines that are based on chemically inactivated bacteria (Porcilis Glässer^®^ – MSD, Suvaxyn Respifend^®^ MH/HPS – Zoetis, Ingelvac^®^ HP-1 – Boehringer Ingelhein, Parapleuro Shield^®^ - Novartis, Hiprasuis^®^ Glässer – Hipra, Rhinanvac Cerdos – Syva). The underlying premise for these vaccines is that disease is caused by a limited number of virulent strains so that vaccines prepared from the predominant disease strains will prevent infection. However, in spite of this selection of available vaccines there are continued outbreaks of Glässer’s disease occurring worldwide. The protection induced by inactivated whole cell vaccines is thought to be directed against the extracellular capsular polysaccharide which is very specific, thus little cross-protection is anticipated against strains expressing other capsular types. This mis-matching of capsular type between disease isolate and vaccine strain is likely a major factor for continued outbreaks of disease. For example, 142 of 278 outbreak cases occurring in 9 Brazilian states in 2015 and 2016 were in vaccinated animals (data from our diagnostic service, unpublished). It is clear that alternate vaccine approaches need to be considered that have the potential for providing broad cross-protection against strains that may vary in the composition of their surface antigens.

The rationale for targeting the surface transferrin receptor for development of a vaccine against *H. parasuis* is based on the observation that it is present in all strains and proposed to be essential for survival on the upper respiratory tract, similar to what has been demonstrated with the human pathogen, *Neisseria gonorrhoeae*, in a human male infection model^23^. In addition, both TbpA and TbpB are essential for disease causation in a porcine infection model with *A. pleuropneumoniae*^24^ suggesting that Tf is the predominant source of iron on the mucosal surface where the aerosolized bacteria are first established and within tissues during progression of infection. The prototypical Tf receptor is present in a wide range of vertebrate species^25^ and the exquisite host specificity of the receptor proteins^26^ has been shown to be a consequence of selective pressures imposed by the bacterial receptor^27^, implying that the host specificity has evolved over relatively long time periods. Since these species reside exclusively in the upper respiratory or genitourinary tracts of their host, the Tf receptors obviously evolved as a mechanism for acquisition of iron on the mucosal surface. The observation that sequence variation in TbpB largely occurs in the Tf binding region of the N-terminal lobe^28–30^ despite the fact that TbpBs bind to the same region of Tf ^21,31–33^, suggest that the different phylogenetic clusters of TbpB variants evolved to provide immunologically distinct binding variants to avoid broad cross-protection by recognition of a conserved binding interface. Thus, efficient exchange of pre-existing variant genes through natural transformation^22^ is likely the primary mechanism by which *H. parasuis, A. pleuropneumoniae* and *A. suis* avoid immune responses against the Tf receptors, suggesting that by targeting the common pool of variant TbpBs, it may be possible to develop a single vaccine against all three pathogens^21^.

The disappointing results in a human Phase I trial for a TbpB-based vaccine against *Neisseria meningitidis*^34^, after promising results in experiments in mice and rabbits^35^, prompted us to question whether the specific binding of host Tf to TbpB interfered with the immune response in humans. This was the impetus for our structure-based approach for designing non-binding mutants of TbpBs from porcine pathogens that could be tested directly in the native host. Thus, we generated site-directed mutant proteins with dramatic loss in binding of porcine Tf (i.e. from 13 nM to 3.6 µM) with no significant alteration in protein structure and tested one mutant (Y167A) in an *H. parasuis* infection model^18^. The enhanced protection provided by the Y167A mutant protein compared to the wild-type protein (6/6 vs 3/6 survived) provided strong support for our hypothesis and strategy for generating improved TbpB antigens. The enhanced protection that this mutant protein provided against the challenge strain, the reference serovar 5 Nagasaki strain, was even more notable considering that only 1/5 pigs survived in the group vaccinated with the commercial serovar 5 vaccine (Porcilis Glässer^®^ – MSD). Additionally, the recognition that this experiment represents a heterologous challenge with respect to TbpB variants (Fig. 1), provides strong support for the proposal for a broadly cross-protective vaccine with antigens derived from a few representative TbpB variants.

In two published studies the protection conferred by the Porcilis Glässer^®^ vaccine against challenge with the Nagasaki strain ranged from being very effective^1^ to ineffective^18^, which we attributed to inadvertently selecting a more virulent colony for the second challenge experiment. This prompted us to switch from selecting single colonies for preparation of challenge inoculum to adopting a protocol for preparing stocks (which contain the whole diversity of clones isolated) for subsequent challenge experiments by passing strains through pigs and using frozen aliquots to start cultures for the challenge experiments.

The current study was initiated to determine whether the lack of Tf binding was sufficient to ensure that a mutant protein would provide superior protection. Since our ongoing sequence analyses (Fig. 1) revealed that the prior study had in fact been a heterologous challenge experiment, it was important to determine whether we could simply generate and use non-binding mutants from the other phylogenetic clusters to generate a broadly cross-protective vaccine. To address this question, we decided to compare the Y167A mutant with the W176A mutant protein that was quite similar in its biochemical and binding properties (3.6 µM vs 0.9 µM) and test them against a reference serovar 7 strain, a serovar currently of concern in Brazil and China. This strain contains the same TbpB variant from which the mutant proteins were derived. The observation that we were able to achieve substantial disease with relatively low inoculum of the reference serotype 7 strain (Fig. 8A), in contrast to previous reports in the literature^36^, could be a reflection that there may be variation in virulence of bacteria within a population and that one must be careful with conclusions regarding disease potential of particular serovars.

In this study, we demonstrated that immunization with the Y167A mutant protein provided substantial protection against challenge with the reference serovar 7 (SV7) *H. parasuis* strain 174 whereas none of the pigs immunized with the W176A mutant protein survived until the end of the experiment (Fig. 8B). Clearly the results of this study and prior studies^18–20^ strongly support the use of the Y167A for protection against various strains causing Glasser’s disease but indicate that W176A mutant TbpB lacks protective efficacy in this model of invasive disease.

A recent study of the immune response against the W176A mutant protein^20^ as well as the analyses performed in this study (Figs 2–7) did not reveal any major defect that could fully explain the lack of protection conferred by this protein. Immunization with the W176A protein, (i) generates a significant immune response (Figs 2 and 3), (ii) produces antibodies capable of binding to intact cells (Fig. 4), (iii) activates B-cells and T-cells (Fig. 5), and (iv) activates complement (Fig. 6). On the other hand, when we compare the results conferred by this protein with the protein that provided protection (Y167A), differences in antibody titre (Fig. 3), complement activation (Fig. 6) and IgG avidity index can be detected, however, why the W176A mutant has induced this lower particular response is not obvious. Although we cannot exclude the possibility that under physiological conditions the W176A mutant would be less stable or more susceptible to proteases there were no observed differences in stability in laboratory experiments and the fact that we were able to crystalize the intact protein for structural studies suggests that this may be unlikely. In summary, the results in this study demonstrate that the structure-based protein engineering approach has produced some promising antigens with improved immunological properties that bode well for developing a cross-protective vaccine against three related porcine pathogens, but that it is not currently possible to predict how effective they will be without testing their efficacy.

Although this study is focused on development of a TbpB-based vaccine against *H. parasuis* and possibly extending it to two other porcine pathogens, the study has much broader implications for vaccine development against important pathogens of humans and food production animals. The ability to directly perform experiments in food production animals provides an expensive but feasible option for ultimately developing and testing improved antigens, but hopefully innovative new approaches will be developed to reduce the costs so that it is within reach of research scientists at academic institutions. Although the development of a broadly cross-protective vaccine against the three porcine pathogens would provide ‘proof of concept’ for the development of similar vaccines against important human pathogens, implementation will require innovative approaches that can identify the antigens that will be effective at disease prevention. A transgenic mouse line expressing human factor H has been used to identify a mutant factor H binding protein that is predicted to be a more efficacious vaccine antigen^37,38^ and a transgenic mouse line expressing human CEACAM1^39^ has provided a colonization model for *N. meningitidis*, providing examples of the types of approaches that could be used for testing candidate antigens and vaccine formulations. The ideal situation would be to develop approaches that can be validated during development of a porcine vaccine, that could be applied to development of human vaccines, thus having greater confidence in their predictive capabilities.

## Material and Methods

### Strains and growth conditions

*H. parasuis* strain 174, the reference strain for serovar 7 (SV7), which was isolated from the nose of a healthy pig^36^ was selected for the challenge experiment. *H. parasuis* was grown on chocolate plate or in PPLO [pleuropneumonia-like organisms (Oxoid, USA)] supplemented with 60 µg/mL nicotinamide adenine dinucleotide (β-NAD, Sigma-Adrich, USA) and 2.5 mg/mL D-glucose (Sigma-Aldrich, USA) and incubated at 37 °C in presence of 5% CO_2_. *Escherichia coli* strains TOP10 (Invitrogen, USA) and ER 2566 (NEB, USA) were grown in LB broth or agar with ampicillin (100 µg/ml) to select for recombinant plasmids. *E. coli* strain ER2566 was cultured in auto-induction media^40^ containing ampicillin (100 µg/ml) (Sigma-Aldrich, USA) for protein production.

### Sequencing and phylogenetic analysis

The overall diversity of TbpB protein sequences from *H. parasuis*, *A. pleuropneumoniae*, and *A. suis* was analyzed with an updated phylogenetic tree. Sequencing of *tbpB* genes was performed as previously described^21^ and the updated tree contains 23 new TbpB protein sequences, in addition to the 59 sequences originally published by Curran, *et al*.^21^. Complete TbpB sequences were manually assembled from the forward, internal, and reverse reads. The mature TbpB sequences were determined using SignalP 4.0 (http://www.cbs.dtu.dk/services/SignalP/)^41^ and alignments were generated using MAFFT v7 (https://mafft.cbrc.jp/alignment/server/)^42^. Maximum likelihood phylogenetic tree was generated using PhyML^43^ and employing the WAG substitution model^44^ with 100 bootstraps to evaluate branch support. The resulting phylogenetic tree visualizations and annotations were performed using FigTree v1.4.2. Three sequences from a presumed TbpB homologue, unable to bind porcine transferrin, were used to root the phylogenetic tree as previously described^21^.

### Recombinant antigen production

The two genes encoding the Y167A and W176A variants of the Hps SV7 TbpB^5,20,26^ were cloned into a custom T7 expression vector containing an N-terminal polyhistidine tag and transformed into *Escherichia coli* strain ER2566 and induced for expression as previously described^18^. The crude lysates from the cells expressing the recombinant proteins were subjected to Ni-NTA chromatography and then subjected to anion exchange with a Q-Sepharose column to further purify the protein. The fractions from the Q-Sepharose column analyzed by SDS-PAGE as pure, were pooled and buffer exchanged into phosphate buffer saline (PBS) and lyophilized. Prior to use in the vaccine formulation the protein was hydrated in double distilled water and filter sterilized (0.22 µm).

### Vaccine formulation

Two experimental vaccines were formulated as described in Table 1. Briefly, 17 mL of filtered (0.22 μm) phosphate buffer [PBS: 137 mM NaCl (Sigma-Aldrich, USA), 2.7 mM KCl (Sigma-Aldrich, USA), 10 mM Na_2_HPO_4_ (Sigma-Aldrich, USA), 1.8 mM KH_2_PO_4_ (Sigma-Aldrich)] pH 8.0 containing 2 mg of recombinant protein were mixed with 3 mL of Montanide Gel 01 (Seppic, France). Stable preparations were obtained by stirring (500 rpm/min) the adjuvant slowly into the antigenic phase, at 4 °C overnight. Vaccines were packaged into 25 mL amber glass bottle, sealed, and stored at 6–8 °C. The vaccine sterility evaluations were performed by inoculating 2 mL of the vaccines in 20 mL of PPLO media supplemented with NAD and D-glucose. The culture was keep at 37 °C, with shaking (200 rpm/min) for 7 days and the turbidity of the cultures were evaluated for 30 days.

### Vaccine safety evaluation in mouse

Six female Balb/c mice of 8 weeks of age were randomly divided into 3 pairs and housed in separated cages. Animals were maintained on a 12:12-hours light:dark cycle with water and sterilized feed *ad libitum*. Each pair were immunized subcutaneously (0.2 mL) with the vaccines listed in the Table 1. After the injection, the animals were clinically evaluated, twice daily during the first 3 days and after, once until the end of one week.

### *H. parasuis* strain 174 challenge dose testing

Six conventional male piglets (Large-White × Landrace) of 42 days of age, negative for *H. parasuis* [Microbiologically: upper respiratory tract free of *H. parasuis* confirmed by culturing on agar chocolate containing 64 μg/mL of bacitracin (Sigma-Aldrich, USA) as recommended by Miani, *et al*.^45^ and PCR from swab using the primers HPS_219690793 as described by Howell, *et al*.^3^. Serologically: two *in house* ELISAs a) based on whole *H. parasuis* SV1, SV4, SV5, SV7, SV12 and b) based on purified Y167A- TbpB] were acquired from the pig farm at the University of Passo Fundo and housed in the Unit for Vaccine Testing and Experimental Infection of Swine (biosafety level 2) of the same institution. After 7 days of acclimatizing to the facility, the animals were ear tagged, weighted and divided into two groups of 3.

The challenge inoculum was prepared by inoculating an aliquot of *H. parasuis* 174 reference strain (SV7) previously passaged in pigs into supplemented PPLO broth (NAD and D-glucose) and incubated at 37 °C with shaking (250 rpm/min) until an optical density of 0.4 at 600 nm was reached. The bacteria were washed twice with PBS pH 7.2 and quantified by Flow Cytometry using a FACSVerse Cytometer (Becton Dickinson, USA) equipped with a 488 nm blue laser, 640 nm red laser and flow sensor (used for volumetric measurement). Two samples containing 10^7^ and 10^8^ *H. parasuis* were adjusted with RPMI 1640 (Invitrogen, USA) to a final volume of 2 mL.

Piglets were challenged by the intratracheal route as previously described by Frandoloso^1^ with a minor modification in the anesthetic protocol; a cocktail of 0.3 mg/kg of acepromazine (Syntec do Brasil, Brazil), 0.3 mg of midazolam (Laboratório Teuto Brasileiro, Brazil) and 15 mg/kg of Ketamine (Ceva Santé Animale, Brazil) was administered by the intramuscular route. After the challenge the rectal temperatures and clinical signs as weakness, apathy, cough, limping, lack of coordination was monitored every 12 hours during the first 7 days post challenge.

### Colostrum-deprived pig production, immunization and experimental challenge

Fifteen colostrum-deprived piglets (DB Genética Suína, Brazil) were used in this experiment. The animals were acquired from a commercial high health farm with no record of *H. parasuis* infection. The sow parturition was carried out as described by Huang, *et al*.^46^ and the newborn piglets were allocated into the transportation cages and transferred to the Unit for Vaccine Testing and Experimental Infection of Swine. The feed strategy used in this study was based in the protocol described by Huang, *et al*.^46^ with minor modification; here, the liquid diet was based on bovine-colostrum pasteurized (60 °C for 1 hour) during the first 10 days and bovine pasteurized milk supplemented with 10% of Mig Lac Instant Plus (Mig-PLUS^®^, Brazil) until day 21. Semi-solid diet was adopted using a commercial pig feed (Mig Mamy PAP, Mig-PLUS^®^) dissolved in bovine milk until day 28 followed by dry feeds until the end of this study [Mig Mamy LAC (day 36 to 49) and Mig Mamy Ini (day 50 to the end)].

Twenty-eight days after birth, piglets were weighed, ear tagged, divided into 3 homogeneous groups of 5 pigs (Table 1) and moved to a common infection room (without physical separation), equipped with plastic floor, temperature control (set at 22 °C), humidity control (70%), microbiological filter (GSI, USA), air renovation (each 5 minutes).

On day 42, the piglets were immunized as described in Table 1 and 14 days after the second immunization (day 77) all pig were anesthetized as described above and challenged by intratracheal injection of 10^7^ *H. parasuis* strain 174. The experiment followed the guidelines of the Brazilian College of Animal Experimentation and was approved by the institutional Committee for Ethical Use of Animals (protocol no. 018/2016).

### Clinical evaluation

Rectal temperatures and other clinical signs (such as weakness, apathy, limping, sneeze, cough, dyspnea, lack of coordination, and/or loss of appetite) were monitored every 12 h during the first 7 days post challenge and, after that, once a day until the end of the study.

### Vaccine immunogenicity analysis

To quantify the antibody production during the immunization process, pig antisera from the groups Y167A-TbpB and W176A-TbpB were analyzed in an Indirect ELISA using plates coated with 1 μg/well of the homologous proteins. The qualitative sera analysis (sera diluted 1:100) was carried out using samples collected on days 0, 14, 21, 28 and 35 of the immunization protocol. The quantitative sera analysis (sera were serially diluted) was also performed using the sera collected on day 35. Briefly, 100 μl of diluted sera in PBST (PBS 0.05% Tween 20) containing 1% skim milk were added to the wells and incubated for 1 hour at 37 °C. Then, wells were washed 3 times with PBST and 100 μl of goat anti-pig whole IgG peroxidase conjugated (Sigma-Aldrich, USA) diluted 1:5.000 or goat anti-pig IgM peroxidase conjugated (Bio-Rad Laboratories Inc, USA) diluted 1:10.000 were added to the wells and incubated for one hour at 37 °C. The wells were washed again and the enzymatic reaction was developed with 3,3,5,5′-tetramethylbenzidine (Sigma-Adrich, USA) +0.06% H_2_O_2_ (Sigma-Adrich, USA). The plates were then incubated in the dark at 22 °C for 15 minutes and the reaction was stopped by adding 3 N HCl. Plates were read at 450 nm using a Synergy HI plate reader (Bio-Tek, USA). The results of the quantitative analysis were described as endpoint titres which are the reciprocal of the highest dilution that gave a positive OD reading [defined as at least two times greater that the OD values of the negative samples (pigs inoculated with PBS + Gel 01)].

### Measurement of antibody affinity

The functional antibody affinity (avidity) of the pig polyclonal antibodies was assessed by an ELISA assay in presence of different concentrations of ammonium thiocyanate as described by Michaelsen, *et al*.^47^. Dilutions of the pig polyclonal antisera (day 35) against Y167A-TbpB and W176A-TbpB that provided an absorbance of approximately 1 (defined by quantitative ELISA as described above) were chosen for subsequent measurements of the avidity. The predetermined sera dilutions were incubated in an ELISA plate coated with the homologous immunizing antigens or with *H. parasuis* strain 174 (SV7) for 1 hour at 37 °C. The plates were washed 5 times with PBST and then duplicates of increasing concentrations of NH_4_SCN in PBST (0 up to 4 M) were added. After incubation for 30 min at 18 °C, the plates were washed 5 times and the secondary antibody (goat anti-pig whole IgG peroxidase conjugated) was added as described above. The antibody avidity was displayed as an avidity index corresponding to the molar concentration of NH_4_ SCN required for producing a 50% reduction of the absorbency compared with wells that did not have NH_4_SCN added. The avidity assay was performed in two independent experiments and the mean of the values was taken as the avidity index.

### Antibody antigenicity against live H. parasuis

In order to determine the capacity of the antibody response induced by the experimental vaccine to recognize live *H. parasuis* strain 174, a flow cytometry analysis was performed. Bacteria were cultured in PPLO liquid supplemented as described above until an OD of 0.2 was reached. 50 μM of deferoxamine mesylate salt (Sigma-Aldrich, USA) was then added to the culture and was kept in a shaking incubator for approximately 8 hours (OD_600_ ~ 0.7). The bacteria were washed twice with PBS and counted. Serum samples from all immunized piglets were heat-treated (56 °C, 30 min) and aliquots of 10 μl were incubated with 990 μl of FACS buffer containing 1 × 10^6^ bacteria for 1 h at 37 °C. The bacteria were washed three times with PBS and coupled antibodies were detected using fluorescein labeled (FITC) goat anti-pig IgG (AbD Serotec, UK) diluted 1:1000 in PBS with 1% bovine serum albumin (BSA) for 1 h at 37 °C. The bacteria were then washed again three times with PBS and suspended in 200 μl of FACS buffer and analyzed by flow cytometry. The data were analyzed using BD FACSuite™ software (BD Biosciences, USA).

### B and T helper cell characterization

The impact of the vaccination on the B and T helper peripheral lymphocytes was assessed by flow cytometry at five time points during the immunization protocol as illustrated in Table 1. For immunostaining, 10^6^ peripheral blood mononuclear cells (PBMC) isolated as described by Frandoloso, *et al*.^48^ and diluted in FACS buffer (containing 0.01% sodium azide) were used. Double staining was performed using the combinations of monoclonal antibodies (mAbs) 74-12-4 (anti-pig CD4 - phycoerythrin [PE] conjugated, IgG2b; BD Pharmingen, USA) and 5C9 (anti-pig IgM-purified, IgG1). The mAb 5C9 was generous gifts from Domínguez Juncal (INIA, Madrid, Spain). PBMCs were incubated in a V-shaped 96-well microplate (Nunc, USA) with primary mAbs for 30 min at 4 °C and washed in FACS buffer three times. The cells were then incubated for 30 min at 4 °C with anti-isotype antibody (FITC-conjugated rat anti-mouse IgG1, A85-1 clone; BD Pharmingen, USA). After a washing step with FACS buffer, the cells were resuspended in 400 μL of FACS buffer and transferred into 1.5 mL Eppendorf tubes (Axygen, USA), and 7-amino actinomycin D (BD Pharmingen, USA) was added to stain the DNA of dead and damaged cells and exclude it from analysis. A total of 40,000 events were acquired in a FACSVerse flow cytometer. The region to perform the analysis of lymphocyte populations was determined using a forward-scatter versus side-scatter dot plot.

### Classical complement pathway activity

The ability of sera from immunized piglets to activate the classical pathway of complement system were evaluated in a complement depletion assay according to the protocol described by Barasuol, *et al*.^20^ with minor modification. To evaluate the complement activation against *H. parasuis* strain 174, bacteria was grown in iron-restricted media, washed twice with CSAB buffer (210 mM triethanolamine, 180 mM citric acid, 10.5 mM MgCl, 1.8 mM CaCl, 1.3 M NaCl, pH 7.4) and counted. 10^7^ bacteria were mixed with 50 µl of inactivated serum diluted 1:5 in CSAB, 25 µl of guinea pig complement containing 5 units of C’H50 (guinea pig complement giving 50% hemolysis) in a 96-well plate (Nunc, USA), and incubated for 1 h at 37 °C. 25 µl of the hemolytic system comprised of sheep red blood cells (SRBC, 2%) sensitized with rabbit hyperimmune serum was added and incubated for 1 hour at 37 °C. To evaluate the complement activation against soluble antigens, recombinant proteins (Y167A-TbpB and W176A-TbpB) were immobilized on ELISA plates as described in the ELISA subsection. Pig inactivated serum + guinea pig complement was incubated for 1 h at 37 °C and then, the total well volume (75 µl) were transferred to a 96-well plate containing the 2% SRBC and incubate for 1 hour at 37 °C. The plates (both experiments) were then centrifuged (250 × g for 2 min) and the supernatant were harvested and read at 540 nm using Synergy HI plate reader (Bio-Tek, USA) for estimating the amount of lysed sensitized SRBC.

### Statistical analysis

Differences amongst treatments were analyzed by the Kruskal-Wallis or one/two-way ANOVA followed by Tukey or Sidak post-test depending on the data normality assessed by Kolmogorov-Smirnov and Levene tests. The comparative survival analysis illustrated in Fig. 6 was performed by Kaplan-Meier curve analysis. The results are reported as means ± SEM and *P*-values < 0.05 were considered to be significant.

## Electronic supplementary material

Supplementary information
