# Supplementary material for: The amino acid selected for generating mutant TbpB antigens defective in binding transferrin can compromise the in vivo protective capacity
Source: Sci Rep. 2018 May 9;8:7372. doi: 10.1038/s41598-018-25685-1 (PMC5943581; doi:10.1038/s41598-018-25685-1)
Supplement: Supplementary file 1 — Supplementary information [file 41598_2018_25685_MOESM1_ESM.pdf]

The amino acid selected for generating mutant TbpB antigens defective in binding transferrin can compromise the *in vivo* protective capacity

João Antônio Guizzo[a], Somshukla Chaudhuri[b], Simone Ramos Prigol[a], Rong-hua Yu[b], Cláudia Cerutti Dazzi[a], Natalia Balbinott[a], Gabriela Paraboni Frandoloso[a], Luiz Carlos Kreutz[a], Rafael Frandoloso [a*‡], Anthony Bernard Schryvers[b**‡]

[a] Laboratory of Microbiology and Advanced Immunology, Faculty of Agronomy and Veterinary Medicine, University of Passo Fundo, Passo Fundo 99052-900, Brazil.

[b] Department of Microbiology & Infectious Diseases, Faculty of Medicine, University of Calgary, Calgary, Alberta, Canada, T2N 4N1.

* Corresponding author at: University of Passo Fundo, Passo Fundo, Rio Grande do Sul, Brazil, 99052-900.

** Corresponding author at: University of Calgary, Calgary, Alberta, Canada, T2N 4N1.

E-mail addresses: rfran@upf.br (Frandoloso, R) and schryver@ucalgary.ca (Schryvers, AB).

‡ Equal contribution

Supplementary figure S1. Rectal temperatures (ºC) in surviving pigs at different time points (days) after the challenge with *H. parasuis* 174 strain (SV7). The blue lines indicate the normal physiological temperature range for pigs. The rectal temperature was measured once a day, at the same time every day. The ambient room temperature was constant at 22ºC.

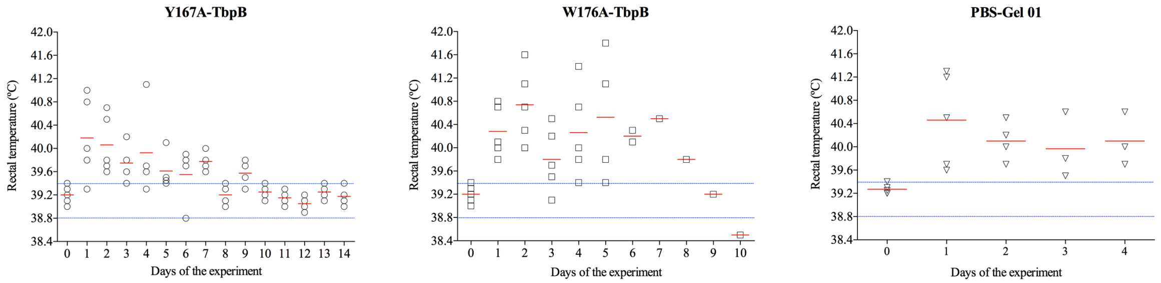

Supplementary Table S1. Clinical evaluation of the pigs challenged with *H. parasuis* strain 174 (serovar 7).

Group	Clinical symptoms	Days of the Experiment and Percentages of Affected Animals														
		0	1	2	3	4	5	6	7	8	9	10	11	12	13	14
Y167A-TbpB	Apathy	•	80%	20%	•	•	•	•	•	•	•	•	•	•	•	†
W176A-TbpB		•	60%	60%	60%	100%	100%	100%	100%	100%	100%	100%	†			
PBS		•	•	80%	100%	100%	†									
Y167A-TbpB	Coughing	•	•	•	•	•	•	•	•	•	•	•	•	•	•	†
W176A-TbpB		•	•	20%	•	•	•	•	•	•	•	•	†			
PBS		•	•	•	•	•	†									
Y167A-TbpB	Dyspnea	•	•	20%	•	•	•	•	•	•	•	•	•	•	•	†
W176A-TbpB		•	•	40%	60%	20%	100%	100%	100%	100%	100%	100%				
PBS		•	•	•	75%	100%	†									
Y167A-TbpB	Lameness	•	•	20%	•	•	•	•	•	•	•	•	•	•	•	†
W176A-TbpB		•	20%	40%	60%	60%	100%	100%	100%	100%	100%	100%	†			
PBS		•	•	40%	100%	100%	†									

• No symptoms detected.

† End of the experiment for each group [where 100% of animals have died as a consequence of the challenge (W176A-TbpB and PBS-Gel 01) or were sacrificed at the end of this study (Y167A-TbpB)].
